# Mobile App to Enhance Patient Activation and Patient-Provider Communication in Major Depressive Disorder Management: Collaborative, Randomized Controlled Pilot Study

**DOI:** 10.2196/34923

**Published:** 2022-10-27

**Authors:** Maggie McCue, Christopher Blair, Ben Fehnert, James King, Francesca Cormack, Sara Sarkey, Anna Eramo, Christopher Kabir, Rasha Khatib, David Kemp

**Affiliations:** 1 Takeda Pharmaceuticals USA, Inc Lexington, MA United States; 2 Cognition Kit Cambridge United Kingdom; 3 Ctrl Group London United Kingdom; 4 Cambridge Cognition Cambridge United Kingdom; 5 Lundbeck LLC Deerfield, IL United States; 6 Advocate Research Institute Advocate Health Care Downers Grove, IL United States; 7 Advocate Aurora Health Downers Grove, IL United States

**Keywords:** depression, major depressive disorder, depression management, patient engagement, patient satisfaction, mobile app, patient-reported outcomes, mobile phone

## Abstract

**Background:**

Enhanced patient-provider engagement can improve patient health outcomes in chronic conditions, including major depressive disorder (MDD).

**Objective:**

We evaluated the impact of a digitally enabled care mobile app, Pathway, designed to improve MDD patient-provider engagement. Patients used a mobile interface to assess treatment progress and share this information with primary care providers (PCPs).

**Methods:**

In this 52-week, real-world effectiveness and feasibility study conducted in primary care clinics, 40 patients with MDD who were recently prescribed antidepressant monotherapy were randomized to use a mobile app with usual care (20/40, 50%) or usual care alone (20/40, 50%). Patients in the app arm engaged with the app daily for 18 weeks; a report was generated at 6-week intervals and shared with the PCPs to facilitate shared treatment decision-making discussions. The patients discontinued the app at week 18 and were followed through year 1. Coprimary outcome measures, assessed via research visits, included change from baseline in the 13-item Patient Activation Measure (PAM-13) and 7-item Patient-Provider Engagement Scale scores at week 18. Additional outcome measures included depression severity (9-item Patient Health Questionnaire [PHQ-9]) and cognitive symptoms (5-item Perceived Deficits Questionnaire–Depression).

**Results:**

All 37 patients (app arm: n=18, 49%; usual care arm: n=19, 51%) who completed the 18-week follow-up period (n=31, 84% female, mean age 36, SD 11.3 years) had moderate to moderately severe depression. Improvements in PAM-13 and PHQ-9 scores were observed in both arms. Increases in PAM-13 scores from baseline to 18 weeks were numerically greater in the app arm than in the usual care arm (mean 10.5, SD 13.2 vs mean 8.8, SD 9.4; *P*=.65). At 52 weeks, differences in PAM-13 scores from baseline demonstrated significantly greater improvements in the app arm than in the usual care arm (mean 20.2, SD 17.7 vs mean 1.6, SD 14.2; *P*=.04). Compared with baseline, PHQ-9 scores decreased in both the app arm and the usual care arm at 18 weeks (mean 7.8, SD 7.2 vs mean 7.0, SD 6.5; *P*=.73) and 52 weeks (mean 9.5, SD 4.0 vs mean 4.7, SD 6.0; *P*=.07). Improvements in 7-item Patient-Provider Engagement Scale and WHO-5 scores were observed in both arms at 18 weeks and were sustained through 52 weeks in the app arm. Improvements in WHO-5 scores at 52 weeks were significantly greater in the app arm than in the usual care arm (41.5 vs 20.0; *P*=.02).

**Conclusions:**

Patients with MDD will engage with a mobile app designed to track treatment and disease progression. PCPs will use the data generated as part of their assessment to inform clinical care. The study results suggest that an app-enabled clinical care pathway may enhance patient activation and benefit MDD management.

**Trial Registration:**

ClinicalTrials.gov NCT03242213; https://clinicaltrials.gov/ct2/show/NCT03242213

## Introduction

### Background

Major depressive disorder (MDD) is among the most common mental health disorders in the United States, with a lifetime prevalence of up to 20% [1]. Although MDD is a chronic and recurrent disorder that frequently requires long-term treatment with antidepressants [2], the rates of nonadherence and premature discontinuation of antidepressant therapy are high and associated with worse clinical outcomes [3-5]. In addition, the impact of nonadherence on clinical outcomes subsequently translates to increased medical and total health care use [3]. In the United States and across the world, most patients with depression are treated by primary care providers (PCPs) [6,7]. Consequently, nearly 10% of all primary care visits are related to depression [8]. Given the recent decline in the number of psychiatrists practicing in the United States, the demand for MDD care in primary care likely will continue to surge [9].

Effective management of MDD in primary care requires a systematic approach to diagnosis, patient education, treatment, close follow-up, and a commitment to adjusting care or consulting with specialists when needed [8,10]. One systematic approach to improving MDD outcomes is measurement-based care, which involves the use of rating scales to monitor symptoms, adherence, and side effects combined with guideline-dependent resources to inform treatment [11]. However, increasing time constraints and the infrequency of visits in primary care settings may make it difficult for clinicians to practice measurement-based care and fully engage with patients with MDD [7,11]. Consequently, patients with MDD and other chronic conditions may benefit from additional support and increased engagement with their PCPs [8,12].

One proposed strategy to increase patient engagement, improve patient-provider communication to facilitate MDD management, and improve patient outcomes in primary care is the use of mobile health apps and digital platforms [13,14]. To improve patient-provider engagement, apps need to be easy to use and easily integrated into the workflow of traditional clinical care and care teams [15]. Currently available apps supporting MDD management are heterogeneous in features and quality, possibly because of the absence of standards governing their development, evaluation, and use [16]. Moreover, the number of apps developed for depression exceeds the number of studies that have demonstrated their efficacy and feasibility. Although few apps have the ability to transmit data directly to PCPs, apps that feature the active involvement of mental health professionals may also increase patient engagement more than presentation enhancements of the technology platform [17]. Digital tools that can share data with PCPs may enable PCPs to more easily and efficiently embrace measurement-based care.

The development of any mobile health and information technology tool, including those supporting MDD management, may benefit from collaboration between industry, app developers, and the health care team representing large health care systems [18]. Research has demonstrated that involving target users and stakeholders in the development of such tools yields a higher acceptance of apps by clinicians. To help meet the needs of PCPs and patients with MDD and improve patient-provider engagement, Takeda, Lundbeck, and Advocate Aurora Health (AAH) worked together with software developers (Ctrl Group, Fora Health, and Cognition Kit) to develop the patient app and care team view of the patient data. The process followed user-centered design principles, in which the designs and development were iterated based on user input and feedback. The app enables patients to track their symptoms, monitor their treatment progress, and share data collected by the app with their care team.

### Objectives

The primary objective of this study was to determine whether the addition of the Pathway mobile app to usual clinical care improves patient-provider engagement in the management of MDD over an 18-week period. The secondary objectives of the study were to evaluate the impact of the Pathway mobile app on changes in certain patient-reported outcomes, including self-reported clinical depression severity (via the 9-item Patient Health Questionnaire [PHQ-9]), cognitive dysfunction (via the 5-item Perceived Deficits Questionnaire–Depression [PDQ-D5]), quality of life (via the 5-item World Health Organization Well-Being Index [WHO-5]), and patient satisfaction, as well as changes in medication and medication adherence. Additional retrospective assessments were also planned to evaluate the impact of the app on measures of health care use 1 year after enrollment.

## Methods

### Study Design

**Figure 1:**
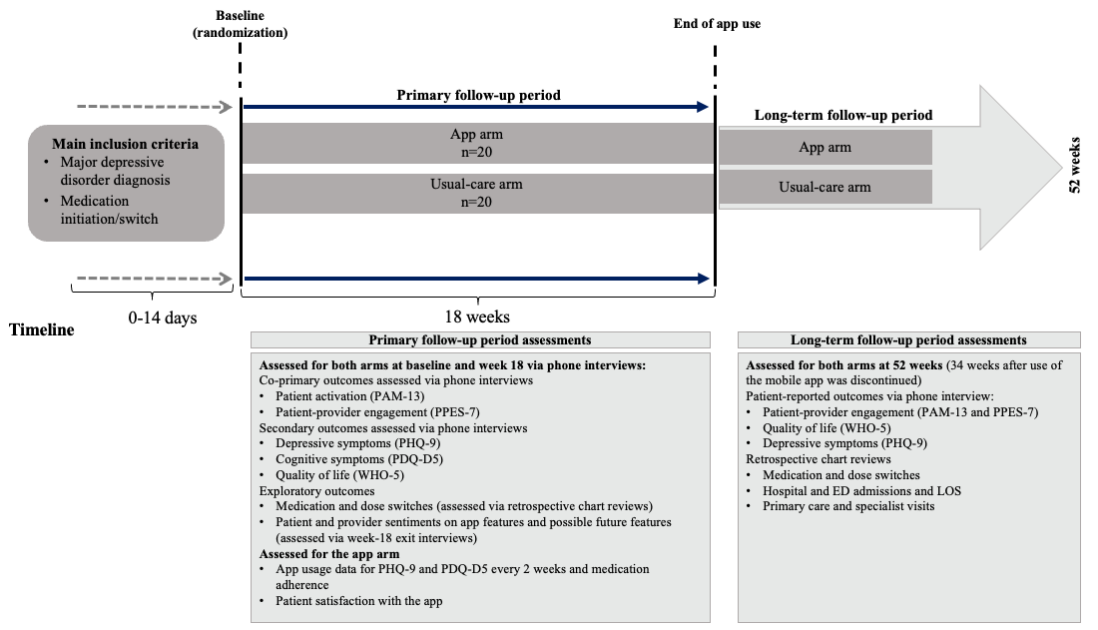
Study design. Long-term follow-up period indicates that no visits, calls, or use of the mobile app occurred during this phase in either treatment arm. The app arm also included usual care. ED: emergency department; LOS: length of stay; PAM-13: 13-item Patient Activation Measure; PDQ-D5: 5-item Perceived Deficits Questionnaire–Depression; PHQ-9: 9-item Patient Health Questionnaire; PPES-7: 7-item Patient-Provider Engagement Scale; WHO-5: 5-item World Health Organization Well-Being Index.

In this randomized controlled pilot study (ClinicalTrials.gov NCT03242213), we enrolled patients diagnosed with MDD who were receiving primary care services at Advocate Health Care, which is now part of AAH. The study took place between July 2017 and January 2019 and involved 4 study sites in suburban and urban settings within metropolitan Chicago, Illinois, including Advocate Medical Group–Huntley, Advocate Medical Group–Hometown Family Medicine, Family Medicine Center in Ravenswood, and Adult Medicine Center in Oak Lawn. On identifying a patient with MDD who met the criteria for study participation, the physician providing care for that patient introduced the study; a designated research study coordinator then explained the study and obtained informed consent. Patients were then randomized to receive either the Pathway mobile app along with usual care or usual care alone. Participating patients were randomized based on the results from a randomized study list created using serially generated random numbers obtained by the study staff using a random number generator. Patients in the mobile app arm were encouraged to engage with the mobile app daily for 18 weeks (Figure 1). At week 18, the use of the mobile app was discontinued. Patients in the usual care arm did not receive study-related interventions.

**Figure 2:**
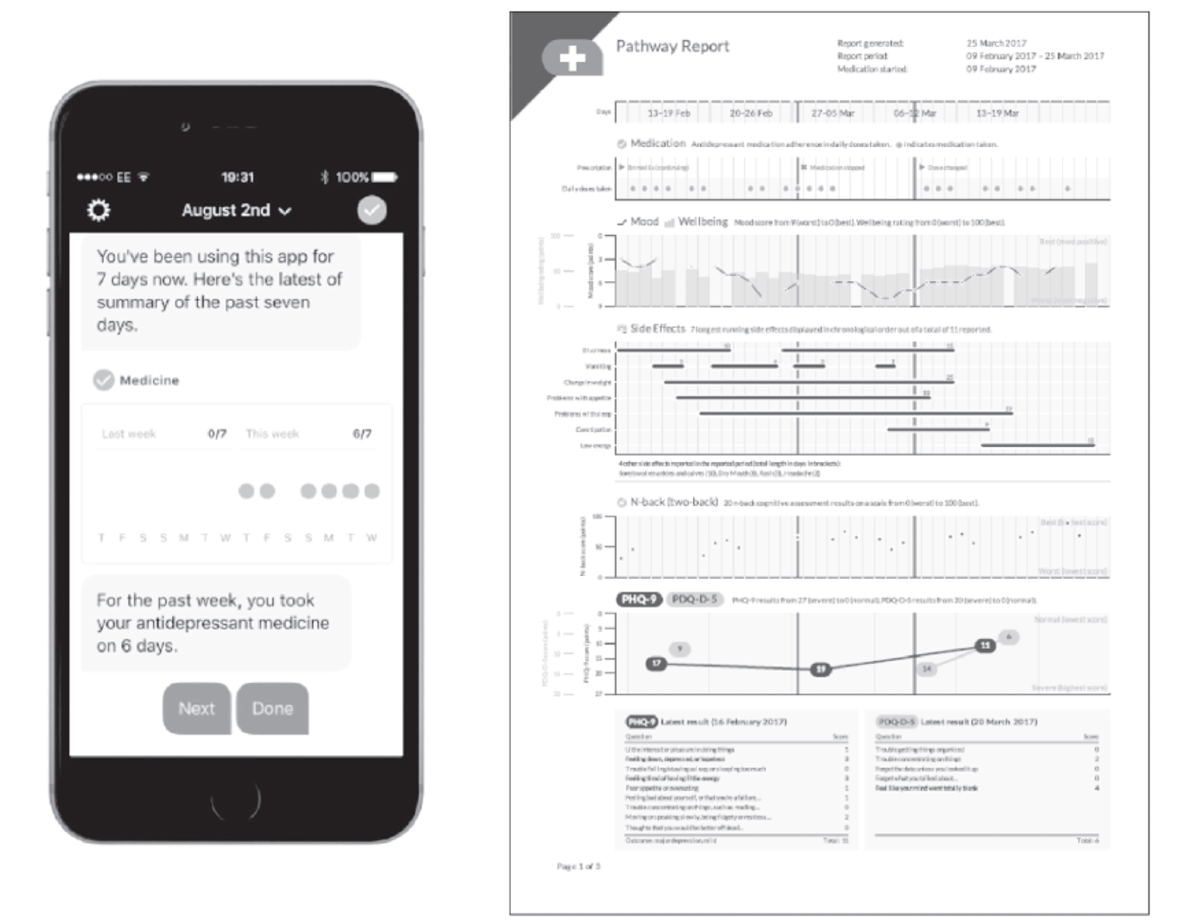
Screenshots of the app and a sample report.

The mobile app was specifically designed to enhance patient-provider engagement, promote shared decision-making, and support measurement-based care in the management of clinical depression. It also provided patients with a way to track changes in clinical depression severity, cognitive symptoms, quality of life, emotional well-being, and adherence to medications; set up medication reminders; and record side-effect experiences. App functions included PHQ-9 and PDQ-D5 assessments conducted every 2 weeks, daily assessments of depression using 2 questions from the PHQ-9 and 1 question from the PDQ-D5, daily assessments of emotional well-being using a visual analog measurement of global well-being on a scale of 0 to 100, and daily cognitive symptoms assessed using the Cognition Kit 2-back test, in which patients indicate whether the symbol (usually an abstract shape) matches the symbol 2 items back [19]. Patients reporting any change in suicidal ideation during PHQ-9 assessments were instructed to contact their health care provider or emergency services immediately because data from the app were not monitored; these instructions were reviewed with patients during the consent process. In addition, patients were able to review a graphical summary of their data (Figure 2), allowing them to review their symptom progression and the treatment’s effectiveness and side effects. A graphical summary of the data was also shared with the care team every 6 weeks to reinforce measurement-based care. No further instructions were provided to the care team.

The single-phase study included 2 follow-up periods: the primary follow-up period and the long-term follow-up period. The primary follow-up period began with randomization and continued through week 18. The long-term follow-up period began after the final visit at week 18 and continued through the 1-year follow-up phone interview (34 weeks after the use of the mobile app was discontinued). At year 1, the results from the follow-up phone interview and extraction of data from patients’ electronic medical records (EMRs) were analyzed to evaluate the residual impact of the app on patient-reported outcomes and health care use.

### Participants

Patients were eligible to participate in the study if they were aged 18 to 70 years, were receiving primary care for MDD at AAH, used an iPhone version 5 or later or a smartphone with an Android operating system, and had an active cellular data plan or regular Wi-Fi access. Patients were also required to have a PHQ-9 score of >5 at baseline, indicating at least mild depression severity [20], and to have initiated monotherapy with a new antidepressant (either a new prescription or a switch from another antidepressant medication) in the previous 0 to 14 days.

Patients were excluded from the study if they had a diagnosis of a major psychiatric disorder other than MDD, were considered to be at imminent risk of hospitalization due to MDD, had been hospitalized due to MDD within 3 months, had a significant risk of suicide or had a previous suicide attempt within 6 months, or had a history of responding only to combination or augmentation therapy in their current depressive episode. Patients for whom the use of antidepressants was contraindicated were not eligible for the study.

### Study Procedures

Eligible patients were randomized to usual care (20/40, 50%) or usual care plus the mobile app (20/40, 50%) for 18 weeks (unblinded for both participants and researchers). An in-person introduction to the app and instructional handouts were provided to the patients at the time of enrollment. Although the use of the mobile app was encouraged, it was not required. Patients assigned to usual care received regular care as needed from their PCP; no specific interventions were mandated. At the end of the long-term follow-up period (1 year), patient-reported outcome measures were collected via follow-up phone calls. Data on health care resource use and medication changes during the long-term follow-up period were collected from medical chart reviews. A quality control committee reviewed the data for adequate completion and integrity.

### Study End Points

Study coprimary, secondary, and exploratory outcomes were assessed at 18 weeks for each arm via in-person research visits and phone interviews. Coprimary outcomes included changes in the 13-item Patient Activation Measure (PAM-13) scale and 7-item Patient Provider Engagement Scale (PPES-7) between baseline and 18 weeks. The PAM-13 scale was developed and validated to assess patient engagement and confidence in self-management of the disease [21,22]. The PAM-13 scores ranged from 0 to 100, with higher values reflecting greater activation. The PPES-7 is an assessment that was developed for this study and has not yet been validated. In the PPES-7, scores range from 7 to 28, with higher values reflecting more engagement.

The secondary outcomes included changes (between baseline and 18 weeks) in depression severity evaluated using the PHQ-9 measure [20], cognitive symptoms measured using the PDQ-D5 scale [23], and quality of life measured using the WHO-5 assessment, a 5-item questionnaire that measures the subjective quality of life. In the WHO-5, scores range from 0 to 100, with higher scores reflecting the best imaginable quality of life [24].

Exploratory outcomes included medication and dose switches, defined as medication switches, dosage changes, medication add-ons, or discontinuations (assessed via retrospective chart review), and patient and provider satisfaction with the care and use of the app. Additional assessments were collected throughout the study in the app arm via app functions such as the PHQ-9, PDQ-D5, emotional well-being, and cognitive symptom assessments. In addition, information on patient satisfaction with the app and app use data were collected from the patients in the app arm.

After the 18-week end-of-study visit, patients and providers were invited to participate in a remote, qualitative, semistructured interview using a digital tool to discuss sentiments on app features and future features. This qualitative tool allowed the interviewer to observe and talk to the patients as they looked at the app’s features on their own devices, record the interview, and capture time-stamped notes.

At the end of the long-term follow-up phase of the study (at year 1), a phone interview was conducted (34 weeks after the use of the mobile app was discontinued) to assess patient-reported outcomes, including patient and provider engagement (using PAM-13 and PPES-7), quality of life (using WHO-5), and depressive symptoms (using PHQ-9). At this time, a retrospective analysis that compared health care use between the app arm and the usual care arm was also conducted. Data were collected on inpatient visits, including depression-related hospitalizations; emergency department (ED) visits; outpatient visits, including visits to PCPs, psychiatrists, behavioral therapy specialists, and other health care providers; and medication and dose switches. Spontaneously reported serious adverse events were also recorded during the study period.

### Statistical Analyses

This was a pilot study, and thus no sample size estimation was conducted. A sample size of 20 patients per group was expected to be sufficient to provide initial information about the potential effects and benefits of the app and the feasibility of its real-world use to inform future larger-scale studies. Patients were included in the analysis based on treatment allocation, and an intent-to-treat analysis was used. As 18-week follow-up data were not available for 3 randomized patients, these patients were dropped from the analysis, and an intent-to-treat analysis (with the exclusion of missing data) was conducted on the remaining population. For the primary and long-term follow-up periods, between-group differences in changes in continuous variables were evaluated using the 2-tailed Student *t* test or Mann-Whitney *U* test. Changes in categorical variables were compared using Fisher exact test or Pearson chi-square test. Two-tailed tests using a significance threshold of *P*<.05 were performed.

A retrospective comparison of health care use was conducted between patients in the usual care arm and those in the mobile app arm using medical record extraction. This comparison included data from the time of consent to 1 year after enrollment for each patient. Health care use was compared between groups for long-term differences at 1 year after each patient’s study enrollment, overall, by cause (depression-related or not) and by category (inpatient via ED, outpatient, or specialty). Sensitivity analysis was used to assess attrition bias among patients lost to follow-up in the long-term follow-up period; these outcomes were used to assess the generalizability of the 52-week results across the original study group. Statistical analysis was performed using SAS software (version 9.4; SAS Institute).

### Ethics Approval

This study (#Y5000249) was approved by the Advocate Health Care Institutional Review Board.

## Results

### Patient Disposition

**Figure 3:**
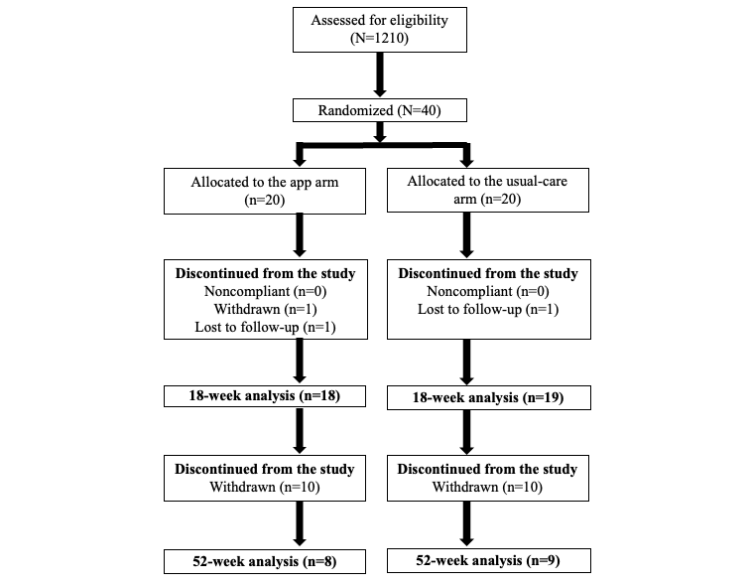
CONSORT (Consolidated Standards of Reporting Trials) diagram.

A total of 40 patients were enrolled, and of them, 37 (93%) completed the 18-week primary follow-up period (Figure 3) and were included in the main analysis based on treatment allocation. In the app arm, 18 (90%) patients completed the primary follow-up period, 1 (5%) withdrew, and 1 (5%) was lost to follow-up. In the usual care arm, 19 (95%) patients completed the primary follow-up period and 1 (5%) was lost to follow-up. At year 1 (the long-term follow-up phase), data were available for 43% (17/40) of patients, including 8 (47%) patients in the app arm and 9 (53%) in the usual care arm.

### Baseline Characteristics

The baseline characteristics of patients who completed 18 weeks of the study are shown in Table 1. Demographic categories were well represented and balanced between each treatment arm, including race (Hispanic, non-Hispanic Black, and non-Hispanic White), geographic area of residence (rural, urban, and suburban), income range, work type, marital status (single, married, or living as a couple; widowed; divorced; or separated), and type of health insurance. The mean age was 33.8 years in the app arm and 38.9 (SD 11.0) years in the usual care arm; 84% (31/37) were women, and 16% (6/37) were men. The mean PHQ-9 score at baseline was 15.3 in the app arm and 14.1 in the usual care arm, indicating moderate to moderately severe depression. Although certain socioeconomic characteristics (eg, education level and annual income) differed between the groups, the groups were similar in terms of MDD severity and treatment history. The baseline demographic characteristics of the 17 patients who completed the long-term follow-up period (the *52-week completers*) were also similar between the groups (Table S1 in Multimedia Appendix 1). When the *52-week completers* were compared with noncompleters with regard to baseline demographics or patient-reported outcomes (PHQ-9, PAM-13, PPES-7, and WHO-5), no statistically significant differences were observed.

**Table 1:** Demographics and baseline characteristics for patients completing 18 weeks.

		App (n=18)	Usual care (n=19)	Total (N=37)
Sex (female), n (%)		14 (78)	17 (90)	31 (84)
Age (years), mean (SD)		33.8 (11.4)	38.9 (11.0)	36.4 (11.3)
Aged ≥45 years, n (%)		2 (11)	5 (26)	7 (19)
**Race or ethnicity, n (%)**				
	Hispanic	8 (44)	6 (32)	14 (38)
	Non-Hispanic Black	2 (11)	5 (26)	7 (19)
	Non-Hispanic White	7 (39)	8 (42)	15 (41)
	Non-Hispanic multiracial	1 (6)	0 (0)	1 (3)
**Employment status, n (%)**				
	Employed full-time	6 (33)	10 (53)	16 (43)
	Employed part-time	5 (28)	3 (16)	8 (22)
	Self-employed	1 (6)	2 (11)	3 (8)
	Not employed	3 (17)	2 (11)	5 (14)
	Student	3 (17)	2 (11)	5 (14)
	Nonworking spouse, retired, or other	2 (11)	2 (11)	4 (11)
Annual income <US $40,000, n (%)		13 (72)	8 (42)	21 (57)
Education (associate’s degree or higher), n (%)		3 (17)	12 (63)	15 (41)
**Number of non–MDD^a^-related medications currently taken, n (%)**				
	0	6 (33)	7 (37)	13 (35)
	1-3	8 (44)	9 (47)	17 (46)
	≥4	4 (22)	3 (16)	7 (19)
PHQ-9^b^, mean (SD)		15.3 (5.1)	14.1 (5.0)	14.7 (5.0)
**Antidepressant use at baseline, n (%)**				
	SSRIs^c^	13 (72)	15 (79)	28 (76)
	Bupropion	2 (11)	3 (16)	5 (14)
	SNRIs^d^	2 (11)	1 (5)	3 (8)
	TCAs^e^, MAOIs^f^, SMSs^g^, or other	1 (6)	0 (0)	1 (3)
**Years on antidepressants, mean (SD)**				
	None	10 (56)	11 (58)	21 (57)
	<1	2 (11)	2 (11)	4 (11)
	≥1	6 (33)	4 (21)	10 (27)
	Unknown	0 (0)	2 (11)	2 (5)

^a^MDD: major depressive disorder.

^b^PHQ-9: 9-item Patient Health Questionnaire.

^c^SSRI: selective serotonin reuptake inhibitor.

^d^SNRI: serotonin and norepinephrine reuptake inhibitor.

^e^TCA: tricyclic antidepressant.

^f^MAOI: monoamine oxidase inhibitor.

^g^SMS: serotonin modulator and stimulator.

### Primary Follow-up Results (18-Week Analysis)

#### Coprimary Outcomes

At week 18, both arms exhibited an increase in patient activation based on the PAM-13 scores (Table S2 in Multimedia Appendix 1), with greater improvement in the app arm than in the usual care arm although this difference was not statistically significant (mean change from baseline 10.5, SD 13.2 vs 8.8, SD 9.4; *P*=.65). Patient-provider engagement improved in both arms based on changes in PPES-7 (Table S2 in Multimedia Appendix 1), with greater improvement in the usual care arm than in the app arm, although again, this difference was not statistically significant (mean change from baseline 1.7, SD 2.7 vs 0.6, SD 3.1; *P*=.27).

#### Secondary Outcomes

Depression severity (as measured by the PHQ-9 score) decreased in both arms (Table S2 in Multimedia Appendix 1). Although the decrease was greater in the app arm (mean change from baseline −7.8, SD 7.2 vs −7.0, SD 6.5), no statistically significant differences were observed between the groups (*P*=.73). In addition, no significant differences in the rates of depression response or remission between groups were reported. Response, defined as a ≥50% decrease in the PHQ-9 score from baseline, was achieved in 56% (10/18) of the patients in the app arm and 58% (11/19) in the usual care arm. Remission, defined as a PHQ-9 score of ≤5, was achieved by 39% (7/18) in the app arm and 44% (8/18) in the usual care arm. Cognitive symptoms (PDQ-D5) and quality of life (WHO-5) improved in both the app arm and the usual care arm (mean change from baseline for PDQ-D5 was −2.6, SD 5.6 vs −5.5, SD 4.3, respectively; *P*=.08; mean change from baseline for WHO-5 was 31.8, SD 19.7 vs 30.7, SD 23.4, respectively; *P*=.88).

#### Exploratory Outcomes

A total of 11% (2/18) of patients in the app arm and 0% (0/18) of patients in the usual care arm switched medications during the study. One serious adverse event (inpatient hospitalization related to depression) was reported in the app arm.

All patients randomized to the app arm (20/20, 100%) completed at least one app assessment during the study period. A majority of patients (12/20, 60%) completed the PHQ-9 and PDQ-D5 assessments biweekly for at least 12 weeks. A total of 70% (14/20) of the app users completed the self-report of medication assessment daily for >100 days.

**Figure 4:**
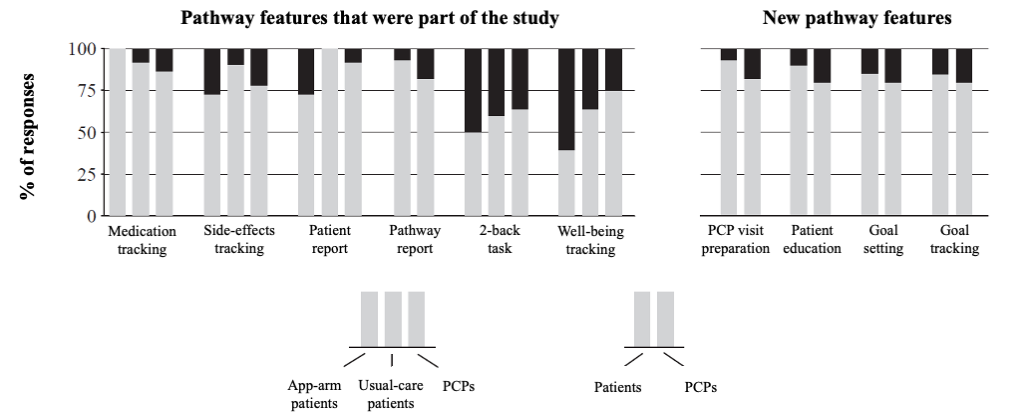
Positive and negative patient and PCP sentiments on Pathway app features (at 18 weeks). Responses were classified as follows: Gray=positive responses: app features were well received; black=negative responses: app features need further work. Data from remote interviews with providers (n=15) and patients (n=20). PCP: primary care provider.

Patient satisfaction in the app arm was high, as shown in the results of the patient satisfaction survey administered at 18 weeks (Table S3 in Multimedia Appendix 1). In remote interviews, >70% of the patients and PCPs provided positive feedback on most of the app’s features and potential new features, including its ability to track medication use and side effects and provide reports (Figure 4). The 2-back task and well-being tracking features scored the lowest, highlighting the need to update these features during the next iteration.

### Long-term Follow-up Results (1-Year Analysis)

#### Patient-Reported Outcomes

At year 1, a significant increase in patient activation (PAM-13) was observed for patients in the app arm (Table S2 in Multimedia Appendix 1), with a greater improvement in the app arm than in the usual care arm (mean change from baseline 20.2, SD 17.7 vs 1.6, SD 14.2; *P*=.04). The quality of life (WHO-5) improved in both arms (mean change from baseline for app vs usual care 41.5, SD 12.3 vs 20.0, SD 19.5), with a significantly greater improvement observed in the app arm (*P*=.02; Table S2 Multimedia Appendix 1). Patients in the app arm experienced improvements in patient-provider engagement as assessed by the PPES-7 although the improvement was not significantly different from that observed in the usual care arm (mean change from baseline for app vs usual care 1.5, SD 2.6 vs 0.1, SD 3.1; *P*=.33). Depression severity (as measured by the PHQ-9 score) decreased in both arms at year 1 (mean change from baseline for app arm vs usual care arm was −9.5, SD 4.0 vs −4.7, SD 6.0; *P*=.07). The longitudinal patient-reported outcomes among patients who completed the 52-week trial are shown in Table S4 in Multimedia Appendix 1.

#### Outcomes From Medical Chart Review

Among the 17 patients assessed during the 1-year follow-up period, there were no inpatient hospitalizations. One patient in the usual care arm visited the ED twice; however, neither visit was considered related to depression. Patients in the usual care arm (n=9) had more outpatient clinic visits to any provider than those in the app arm (n=8; 88 visits vs 49 visits), including visits to PCPs (59 visits vs 38 visits). At 1-year follow-up, 3 patients in each group had a medication change: 1 patient in the mobile app arm and 2 in the usual care arm switched medications; 1 patient in each group had a medication dose change; and 1 patient in the mobile app arm added a new medication to their regimen.

## Discussion

### Principal Findings

The results of this pilot study suggest that the Pathway mobile app may facilitate the systematic use of measurement-based care in MDD management, which can enhance shared decision-making and patient-provider communication, with improved medication adherence and treatment outcomes [10]. The small sample size of this study prevents interpretation based on individual characteristics; however, a larger implementation study is underway (NCT04891224) [25]. Patients in the mobile app arm exhibited a greater change in patient activation (PAM-13) from baseline, with a 10.5-point increase over 18 weeks; this was numerically better than that observed for the usual care arm, although these differences were not statistically significant. The PAM-13 is scored on a scale from 0 to 100, and PAM-13 results are categorized into 4 levels of patient activation: level 1 (0-47.0), which suggests that patients may not yet understand that their role is important; level 2 (47.1-55.1), which indicates that patients lack the confidence or knowledge to take action; level 3 (55.2-72.4), which suggests that patients are beginning to engage in recommended health behaviors; and level 4 (72.5-100), which indicates that patients are proactive about health and engage in many recommended health behaviors [26].

In this study, the mean PAM-13 scores suggested that patients in the app arm, on average, moved from PAM-13 level 3 (58.2) to level 4 (74.2) at the end of the primary follow-up period, whereas patients in the usual care group, on average, started and remained at level 3. A cross-sectional study of patients visiting a primary care clinic reported that every 10-point increase in the PAM-13 score was associated with a 1% reduction in the predicted probability of having an ED visit or being obese and a 1% increase in the predicted probability of having clinical indicators (eg, hemoglobin A1_c_) in the normal range [22]. Taken together, these results may suggest that patients who used the app have a slightly greater likelihood of engaging in proactive health behaviors. In addition, the increase in patient-provider engagement (as measured by the PPES-7 score) noted for both arms may further increase this likelihood by helping patients and clinicians make better care decisions, thus improving the ability of patients to effectively manage their own care [27].

The severity of depression, quality of life, and subjective cognitive symptoms improved in both arms, with no statistically significant differences between groups from baseline to week 18. Although not statistically significant, a trend toward improvement in depressive symptoms (PHQ-9) was observed among the patients in the app arm. Patient-provider engagement also showed small improvements in both arms. These results suggest that a larger study is warranted to determine whether the use of the app is associated with a clinically meaningful improvement in the symptoms of depression or patient-provider engagement.

At year 1, greater improvement in patient activation (PAM-13; *P*=.04) and quality of life (WHO-5; *P*=.02) was observed for patients in the mobile app arm than in the usual care arm, indicating that the app was associated with a long-term impact on patient activation that was sustained for at least 34 weeks after the app was discontinued. Small improvements were observed in both treatment arms with regard to patient-provider engagement (PPES-7) from baseline to week 52. It is possible that the reported improvement at year 1 among patients who completed the 1-year trial may be influenced by attrition bias. However, patients who were lost to follow-up were similar in most baseline characteristics to those who completed the study although they did have higher PHQ-9 scores at baseline and were more likely to have received education beyond high school. The similarity between the populations that completed the study and those that were lost to follow-up suggests that the year 1 results may be generalizable to the original study population.

Moreover, although the overall number of medication changes was similar in both groups at 52 weeks, 2 switches occurred in the app group before the week 18 assessment, with no observed switches in the usual care group in that time frame. In addition, the number of outpatient visits (overall and PCP visits) was greater for patients in the usual care arm than for those in the app arm. These examples may suggest that the app, through improved patient-provider communication, allowed for a more rapid response to changes in patient status while reducing the burden of in-person office visits.

Several systematic reviews and meta-analyses have shown that the use of digital mental health interventions such as apps can aid in the reduction of depressive symptoms, with larger effects seen in patients with more severe symptoms [28,29]. In addition, a randomized clinical trial of a mobile intervention app–based platform, IntelliCare, in primary care patients positive for depression or anxiety demonstrated a greater reduction in symptoms, with sustained changes over a 2-month follow-up period compared with participants in the control arm [30]. Furthermore, a pilot study in 23 women with postpartum depression demonstrated that the enhancement of clinical care with ecological momentary assessment using a wearable device to track daily symptoms, depression, anxiety, and maternal functioning was found to be clinically useful by both study participants and the study clinician [31]. In this study, the digitally enabled care pathway showed sustained effects for up to 1 year (34 weeks after the mobile app intervention was discontinued) on patient-provider engagement, clinical symptoms, quality of life, and resource use. These results confirm the findings of previous studies that demonstrated that app use in patients with MDD and other chronic conditions can have a positive effect on patient adherence, symptoms of depression and anxiety, and patient engagement with therapeutic interventions [13,28-33]. In summary, digital mental health interventions to support clinical decisions and empower shared decision-making may confer solutions to existing barriers and high discontinuation rates observed in psychotherapy.

Although the app users used the app for only 18 weeks, the 34-week follow-up period enabled us to determine whether the benefits of the app were sustainable. The length of the follow-up period is unique in the field of mobile health research on MDD. In fact, a recent review of the effectiveness of apps targeting patients with MDD identified 18 studies evaluating their impact on depression [16], and none of the studies was for >4 months in duration.

Two important strengths of our pilot study were its randomized controlled study design and the long-term 1-year follow-up period. Another strength was that the app was developed and piloted in collaboration with end users in the health care team and cocreated with patient end users. Research has demonstrated that the effectiveness of digital technology resources can depend on the extent to which end users are included as active participants in their design [18]. Moreover, approximately 60% of the study population was Hispanic, non-Hispanic Black, or multiracial, suggesting that the patients included in the study were largely representative of the racial and ethnic diversity observed in the US population.

### Limitations and Future Directions

Potential limitations of the study included its small sample size, which limited our ability to identify statistically significant differences between groups, and the relatively short duration of app use although we were able to maintain follow-up with nearly half of the study’s participants after they discontinued its use. In addition, although patients and providers generally expressed high satisfaction with the app and interest in its features, both groups received limited education on how to use the app or its associated reports. The provision of additional education about the functionality and reporting features of the app might help increase patient-provider engagement and lead to improvements in the overall management of MDD. Embedding the reporting feature into the EMR rather than it being a stand-alone report might also improve the ability of PCPs to make real-time decisions about treatment. Additional work on the Pathway platform informed by the results of this study will help integrate the digitally enabled MDD care pathway into the current AAH system by assessing process and workflow improvements, clinician-patient experiences, collaborative care model enhancements, EMR integration, and efficiencies with other platforms.

Our study data and qualitative insights informed the design of a real-world, prospective, interventional study of the app currently underway at AAH, designed to test the scaling and integration of the Pathway platform, along with educational interventions, at multiple primary care sites (Clinical Trials.gov NCT04891224). The goal of this study was to determine whether the use of the app can improve adherence to measurement-based care practices in primary care to help improve outcomes for patients with MDD.

### Conclusions

This pilot study demonstrated that patients with MDD will engage with a mobile app designed to track treatment and disease progression and that health care providers will use the data generated as part of their assessment to inform care. The study results demonstrate that it is feasible to conduct an innovative app intervention in this diverse patient population with moderate to moderately severe depression. Introducing a customized, cocreated patient app into the care pathway can provide both patients and clinicians with greater details and trend data related to the disease state outside the traditional in-person visit. Enhanced use of patient-reported data within real-world health care settings can help support measurement-based care practices by making patient self-reported data and summaries of the data easy to interpret and easy to access within existing EMR instances.

Although the sample size was small for the long-term follow-up phase of the study, the results of this feasibility study suggest that this digitally enabled MDD clinical care pathway approach may support shared decision-making and help provide sustainable benefits over at least 1 year. The impact of the app on patient activation and MDD management will be further explored in a larger prospective study of its real-world use in patients with MDD.

## Appendix

Multimedia Appendix 1 Key study demographics, patient-reported outcomes, and longitudinal results.

Multimedia Appendix 2 CONSORT-eHEALTH (Consolidated Standards of Reporting Trials of Electronic and Mobile Health Applications and Online Telehealth) checklist.

## Abbreviations

AAH: Advocate Aurora Health
CONSORT-eHEALTH: Consolidated Standards of Reporting Trials of Electronic and Mobile Health Applications and Online Telehealth
ED: emergency department
EMR: electronic medical record
MDD: major depressive disorder
PAM-13: 13-item Patient Activation Measure
PCP: primary care provider
PDQ-D5: 5-item Perceived Deficits Questionnaire–Depression
PHQ-9: 9-item Patient Health Questionnaire
PPES-7: 7-item Patient-Provider Engagement Scale
WHO-5: 5-item World Health Organization Well-Being Index

## Data Availability

The data supporting the findings of this study are included in the published paper (and its supplementary information files). The authors may be contacted for further data sharing. The CONSORT‐eHEALTH (Consolidated Standards of Reporting Trials of Electronic and Mobile Health Applications and Online Telehealth) checklist is available as Multimedia Appendix 2.
