# Supplementary material for: Mobile App to Enhance Patient Activation and Patient-Provider Communication in Major Depressive Disorder Management: Collaborative, Randomized Controlled Pilot Study
Source: JMIR Form Res. 2022 Oct 27;6(10):e34923. doi: 10.2196/34923 (PMC9650572; doi:10.2196/34923)
Supplement: Multimedia Appendix 1 [file formative_v6i10e34923_app1.pdf]

## Multimedia Appendix 1

**Table S1.** Demographics and baseline characteristics of patients who completed 52 weeks

| | App arm (n=8) | Usual-care arm (n=9) | *P* value |
|---|---|---|---|
| Age, y (SD) | 34.6 (6.0) | 42.6 (12.1) | .11 |
| Female, n (%)[a] | 7 (87.5) | 8 (88.9) | .93 |
| **Race and ethnicity, n (%)** | | | .11 |
| Hispanic | 5 (62.5) | 2 (22.2) | |
| Non-Hispanic Black | 0 | 3 (33.3) | |
| Non-Hispanic White | 3 (37.5) | 4 (44.4) | |
| **Geographic area of residence, n (%)** | | | .20 |
| Rural | 2 (25.0) | 0 | |
| Urban | 4 (50.0) | 4 (44.4) | |
| Suburban | 2 (25.0) | 5 (55.6) | |
| **Income range, n (%)** | | | .99 |
| <$40,000 | 5 (62.5) | 5 (55.6) | |
| ≥$40,000 | 3 (37.5) | 4 (44.4) | |
| **Work type, n (%)** | | | .35 |
| Full-time (at least 40 hours per week) | 5 (62.5) | 3 (33.3) | |
| Less than full-time or other[a] | 3 (37.5) | 6 (66.7) | |

[a]Fisher exact test.

**Table S2.** Summary of key patient-reported outcomes at 18 and 52 weeks

| | | | | App | | Usual care | | *P* value |
|---|---|---|---|---|---|---|---|---|
| | | | | Values, n (%) | Values, mean (SD) | Values, n (%) | Values, mean (SD) | |
| **Coprimary outcomes** | | | | | | | | |
| | **PAM-13[a]** | | | | | | | |
| | | **Week 18 analysis** | | | | | | |
| | | | Baseline | 18 (100%) | 56.6 (13.6) | 18 (95%) | 58.6 (11.3) | — |
| | | | Week 18 | 18 (100%) | 67.1 (12.1) | 18 (95%) | 67.4 (15.6) | — |
| | | | Change from baseline to week 18 | — | 10.5 (13.2) | — | 8.8 (9.4) | .65 |
| | | **Week 52 analysis** | | | | | | |
| | | | Baseline | 8 (100%) | 58.2 (10.8) | 9 (100%) | 61.4 (12.5) | — |
| | | | Week 52 | 8 (100%) | 78.4 (18.5) | 9 (100%) | 62.5 (15.9) | — |
| | | | Change from baseline to week 52 | — | 20.2 (17.7) | — | 1.6 (14.2) | .04 |
| | **PPES-7[b]** | | | | | | | |

| | | | | | | | | |
|---|---|---|---|---|---|---|---|---|
| | | **Week 18 analysis** | | | | | | |
| | | | Baseline | 17 (94%) | 22.6 (4.1) | 19 (100%) | 22.9 (1.9) | — |
| | | | Week 18 | 17 (94%) | 23.2 (3.1) | 19 (100%) | 24.6 (2.9) | — |
| | | | Change from baseline to week 18 | — | 0.6 (3.1) | — | 1.7 (2.7) | .27 |
| | | **Week 52 analysis** | | | | | | |
| | | | Baseline | 8 (100%) | 23.5 (3.0) | 9 (100%) | 22.6 (2.9) | — |
| | | | Week 52 | 8 (100%) | 25.0 (2.6) | 9 (100%) | 22.7 (3.1) | — |
| | | | Change from baseline to week 52 | — | 1.5 (2.6) | — | 0.1 (3.1) | .33 |
| **Secondary outcomes** | | | | | | | | |
| | **PHQ-9[c]** | | | | | | | |
| | | **Week 18 analysis** | | | | | | |
| | | | Baseline | 18 (100%) | 15.3 (5.1) | 19 (100%) | 14.1 (5.0) | — |
| | | | Week 18 | 18 (100%) | 7.5 (5.7) | 19 (100%) | 7.1 (5.0) | — |

| | | | | | | | | |
|---|---|---|---|---|---|---|---|---|
| | | | Change from baseline to week 18 | — | −7.8 (7.2) | — | −7.0 (6.5) | .73 |
| | **Week 52 analysis** | | | | | | | |
| | | | Baseline | 8 (100%) | 13.5 (3.3) | 9 (100%) | 13.2 (6.4) | — |
| | | | Week 52 | 8 (100%) | 4.0 (3.2) | 9 (100%) | 8.6 (5.5) | — |
| | | | Change from baseline to week 52 | — | −9.5 (4.0) | — | −4.7 (6.0) | .07 |
| **PDQ-D5[d,e]** | | | | | | | | |
| | **Week 18 analysis** | | | | | | | |
| | | | Baseline | 18 (100%) | 9.7 (4.1) | 19 (100%) | 10.7 (5.4) | — |
| | | | Week 18 | 18 (100%) | 7.2 (4.7) | 19 (100%) | 5.3 (4.8) | — |
| | | | Change from baseline to week 18 | — | −2.6 (5.6) | — | −5.5 (4.3) | .08 |
| **WHO-5[f]** | | | | | | | | |
| | **Week 18 analysis** | | | | | | | |
| | | | Baseline | 18 (100%) | 20.2 (8.3) | 19 (100%) | 29.7 (11.8) | — |

| | | | | | | | | |
|---|---|---|---|---|---|---|---|---|
| | | | Week 18 | 18 (100%) | 52.0 (19.5) | 19 (100%) | 60.4 (20.6) | — |
| | | | Change from baseline to week 18 | — | 31.8 (19.7) | — | 30.7 (23.4) | .88 |
| | | **Week 52 analysis** | | | | | | |
| | | | Baseline | 8 (100%) | 23.0 (6.0) | 9 (100%) | 27.6 (13.2) | — |
| | | | Week 52 | 8 (100%) | 64.5 (9.9) | 9 (100%) | 47.6 (23.5) | — |
| | | | Change from baseline to week 52 | — | 41.5 (12.3) | — | 20.0 (19.5) | .02 |

[a]PAM-3: 13-item Patient Activation Measure.
[b]PPES-7: 7-item Patient-Provider Engagement Scale.
[c]PHQ-9: 9-item Patient Health Questionnaire.
[d]PDQ-D5: 5-item Perceived Deficits Questionnaire–Depression.
[e]PDQ-D5 was not evaluated at week 52.
[f]WHO-5: 5-item World Health Organization Well-Being Index.
—, not applicable.

**Table S3.** Patient satisfaction in the app arm at 18 weeks

| How Satisfied* Are You With… | Median (IQR) |
|---|---|
| How easy the app is to use? | 6.0 (1.0) |
| The app helping you understand the effect of your medication? | 5.5 (1.0) |
| Using the app and the reports generated in addition to your usual care with your doctor? | 6.0 (1.0) |
| The amount of time the app required? | 5.0 (1.0) |

*Satisfaction ranged from 1 (extremely dissatisfied) to 7 (extremely satisfied).
Data per in-person patient interview (n=18).
IQR, interquartile range.

**Table S4.** Longitudinal results of key patient-reported outcomes[a] among patients who completed 52 weeks

| | App (n=8) | | Usual Care (n=9) | |
|---|---|---|---|---|
| **Coprimary outcomes** | n | Mean (SD) | n | Mean (SD) |
| **PAM-13** | | | | |
| Baseline | 8 | 58.2 (10.8) | 8 | 61.4 (12.5) |
| Week 18 | 8 | 74.2 (10.0) | 9 | 70.3 (16.8) |
| Week 52 | 8 | 78.4 (18.5) | 9 | 62.5 (15.9) |
| **PPES-7** | | | | |
| Baseline | 8 | 23.5 (3.0) | 9 | 22.6 (2.2) |
| Week 18 | 8 | 25.0 (3.0) | 9 | 25.2 (3.3) |
| Week 52 | 8 | 25.0 (2.6) | 9 | 22.7 (3.1) |
| **Key secondary outcomes** | | | | |
| **PHQ-9** | | | | |
| Baseline | 8 | 13.5 (3.3) | 9 | 13.2 (6.4) |
| Week 18 | 8 | 4.4 (3.8) | 9 | 8.1 (5.2) |
| Week 52 | 8 | 4.0 (3.2) | 9 | 8.6 (5.5) |
| **WHO-5** | | | | |
| Baseline | 8 | 23.0 (6.0) | 9 | 27.6 (13.2) |
| Week 18 | 8 | 62.5 (19.2) | 9 | 57.8 (22.4) |
| Week 52 | 8 | 64.5 (9.9) | 9 | 47.6 (23.5) |

[a]PDQ-D5 was not evaluated at week 52.

PAM-13, 13-item Patient Activation Measure; PDQ-D5, 5-item Perceived Deficits Questionnaire–Depression; PHQ-9, 9-item Patient Health Questionnaire; PPES-7, 7-item Patient Provider Engagement Scale; WHO-5, 5-item World Health Organization Well-Being Index.
