# Supplementary material for: Mobile App to Enhance Patient Activation and Patient-Provider Communication in Major Depressive Disorder Management: Collaborative, Randomized Controlled Pilot Study
Source: JMIR Form Res. 2022 Oct 27;6(10):e34923. doi: 10.2196/34923 (PMC9650572; doi:10.2196/34923)
Supplement: Multimedia Appendix 2 [file formative_v6i10e34923_app2.pdf]

Multimedia Appendix 2.

CONSORT-EHEALTH checklist (V.1.6.1): Information to include when reporting ehealth/mhealth trials (web-based/Internet-based intervention and decision aids, but also social media, serious games, DVDs, mobile applications, certain telehealth applications)

		Page #	Response
1	1ai	1	Title<br>**"Identify the mode of delivery in the title"**<br>Yes; Title contains words "digitally enabled" as per the requirement
	1aii	n/a	n/a
	1aiii	1	Title<br>**"Mention primary condition or target group in the title"**<br>Yes; "major depressive disorder"
	1bi	2	Abstract, Methods<br>**"Key features, functionalities/components of the intervention"**<br>Yes; "In this 52-week, real-world effectiveness and feasibility study conducted in primary care clinics, in 40 patients with MDD and recently prescribed antidepressant monotherapy were randomized to use of a mobile app…" (lines 32-34)
	1bii	2	Abstract, Methods:<br>**"Clarify the level of human involvement in the abstract"**<br>"..a report was generated at 6-week intervals and shared with PCPs to facilitate shared treatment decision-making discussions" (lines 35-36)
1biii		2	Abstract, Methods<br>Open vs. closed, web-based (self-assessment) vs. face-to-face assessments in abstract:<br>**"Recruitment status (online vs offline)"**<br>"In this 52-week, real-world effectiveness and feasibility study conducted in primary care clinics,…"(lines 32-34)<br><br>**"Web-based vs face-to-face"**<br>"..a report was generated at 6-week intervals and shared with PCPs to facilitate shared treatment decision-making discussions" (lines 35-36)<br><br>**"Clearly say if outcomes were self assessed through questionnaires (as common in web-based trials)"**<br>"Coprimary outcome measures, assessed via research visits, included change from baseline in PAM-13 and PPES-7 at week 18." (lines 37-39)

		Page #	Response
	1biv	2-3	Abstract, Results<br>**"Number of participants in each group"**<br>"Of the 37 patients (app arm, n=18; usual-care arm, n=19) who completed the 18-week primary follow-up period (lines 44-45)<br><br>**"Primary/Secondary outcomes"**<br>"Improvements in PAM-13 and PHQ-9 scores ..”- (lines 46-56)
	1bv	3	Abstract, conclusions<br>"Study results suggest an app-enabled clinical care pathway may enhance…" (lines 59-60)
2	2ai	5-6	Introduction<br>**"Describe the problem and the type of system/solution that is object of the study"**<br>**"Intended for a particular patient population"**<br>**"Goals"**<br><br>"The primary objective of the study was to determine whether the addition of the Pathway mobile app to usual clinical care improves patient-provider engagement in the management of MDD…" (lines 120-129)
	2aii	4-5	Introduction<br>**Scientific background**<br>"Effective management of MDD...with their PCPs" (lines 83-91)<br>"One proposed strategy to increase patient engagement..." (lines 93-95)<br>"Currently available apps…" (lines 97-103)<br><br>**Rationale**<br>"The development of any mobile health and information technology tool… app with their care team" (lines 107-118)
	2b	n/a	**"Specific objectives or hypotheses"**<br>n/a
3	3a	6-7	Methods<br>**"Description of trial design"**<br>Study design (lines 133-160)
	3bi	n/a	**"Bug fixes, Downtimes, Content Changes"**<br>N/a
4	4ai	8	Methods<br>**Computer / Internet literacy**<br>n/a as it is a mobile app "An in-person introduction to the app and instructional handouts were provided to patients at the time of enrollment." (lines 194-195)
	4aii	6, 8	Participants

		Page #	Response
			**Open vs. closed, web-based vs. face-to-face assessments**<br>“In this randomized controlled pilot study (ClinicalTrials.gov NCT03242213), we enrolled patients diagnosed with MDD who were receiving primary care services at Advocate Health Care, now part of AAH...” (ln 134-139)<br><br>“Study coprimary, secondary, and exploratory outcomes were assessed at 18 weeks for each arm via in-person research visits and phone interviews.” (lines 204-205)
	4aiii	6, 8	**Information given during recruitment**<br>“Upon identifying a patient with MDD who met criteria for study participation, the physician providing care for that patient introduced the study; a designated research study coordinator then explained the study and obtained informed consent.” (lines 140-143)<br><br>“An in-person introduction to the app and instructional handouts were provided to patients at the time of enrollment.” (lines 194-195)
	4bi	6	**“Clearly report if outcomes were (self-)assessed through online *questionnaires*”**<br>“Study coprimary, secondary, and exploratory outcomes were assessed at 18 weeks for each arm via in-person research visits and phone interviews.” (lines 204-205)
	4bii	n/a	**“Report how institutional affiliations are displayed”**<br>n/a
5	5i	5, 21, 22	**“Mention names, credential, affiliations of the developers, sponsors, and owners”**<br>**“**Takeda, Lundbeck, and Advocate Aurora Health (AAH) worked together with software developers (Ctrl Group/Fora Health & Cognition Kit) to develop the patient app and care team view of the patient data.” (lines 112-114)<br><br>“Authors’ Contributions” (lines 521-533)<br><br>“Conflicts of Interest” (lines 541-548)
	5ii	5	**“Describe the history/development process”**<br>“To help meet the needs of PCPs and patients with MDD..., Takeda, Lundbeck, and Advocate Aurora Health (AAH) worked together with software developers (Ctrl Group/Fora Health & Cognition Kit) to develop the patient app and care team view of the patient data.” (ln 111-114)
	5iii	6-7	**“Revisions and updating”**<br>“App functions included PHQ-9 and PDQ-D5 assessments conducted every 2 weeks, daily assessments of depression using 2 questions from PHQ-9 and 1 question from PDQ-D5, ...” (lines 155-163)
	5iv	8	**Provide information on quality assurance methods**<br>“A quality control committee reviewed the data for adequate completion and integrity.” (lines 200-201)
	5v	6-7	**“Ensure replicability by publishing the source code, and/or providing screenshots/screen-capture video, and/or providing flowcharts of**

		Page #	Response
			**the algorithms used"**<br>"App functions included PHQ-9….to reinforce measurement-based care" (lines 155-167)
	5vi	n/a	**"Digital preservation"**<br>**n/a**
	5vii	5	"**Access**"<br>"In this randomized controlled pilot study (ClinicalTrials.gov NCT03242213), we enrolled patients diagnosed with MDD who were receiving primary care services at Advocate Health Care, now part of AAH" (lines 134-136)
	5viii	5, 6-7	**"Describe mode of delivery, features/functionalities/components of the intervention and comparator, and the theoretical framework"**<br>"To help meet the needs of PCPs and patients with MDD and improve patient-provider engagement, Takeda, Lundbeck, and Advocate Aurora Health (AAH) worked together with software developers (Ctrl Group/Fora Health & Cognition Kit) to develop the patient app and care team view of the patient data." (lines 111-114)<br><br>"The mobile app was specifically designed to enhance patient-provider engagement….. was also shared with the care team every 6 weeks to reinforce measurement-based care." (lines 151-164)
	5ix	6	**"Describe use parameters"**<br>"Patients in the mobile app arm were encouraged to engage with the mobile app daily for 18 weeks (Figure 1). At week 18, use of the mobile app was discontinued. Patients in the usual-care arm received no study-related interventions." (lines 147-149)
	5x	8	**"Clarify the level of human involvement"**<br>"An in-person introduction to the app and instructional handouts were provided to patients at the time of enrollment. Although use of the mobile app was encouraged, it was not required. Patients assigned to usual care received regular care as needed from their PCP; no specific interventions were mandated." (lines 194-197)<br><br>"Study coprimary, secondary, and exploratory outcomes were assessed at 18 weeks for each arm via in-person research visits and phone interviews." (lines 204-205)<br><br>"At the end of the long-term follow-up period (at 1 year), patient-reported outcome measures were collected via follow-up phone calls." (lines 197-199)
	5xi	6	**"Report any prompts/reminders used"**<br>**"**App functions included PHQ-9 and PDQ-D5 assessments conducted every 2 weeks, daily assessments of depression using 2 questions from PHQ-9 and 1 question from PDQ-D5, daily assessments of emotional well-being using a visual analog measurement of global well-being on a scale of 0 to 100, and daily cognitive symptoms assessed with the Cognition Kit 2-back test" (lines 155-159)
	5xii	8	**"Describe any co-interventions (incl. training/support)":**<br>"An in-person introduction to the app and instructional handouts were provided to patients at the time of

		Page #	Response
			enrollment.” (lines 194-195)
6	6ai	n/a	**“If outcomes were obtained through online questionnaires, describe if they were validated for online use and apply CHERRIES items to describe how the questionnaires were designed/deployed”**<br>n/a
	6aii	15	**Describe whether and how “use” (including intensity of use/dosage) was defined/measured/monitored (logins, logfile analysis, etc.)**<br>“All patients randomized to the app arm (n=20) completed at least one app assessment during the study period. A majority of patients (60%) completed the PHQ-9 and PDQ-D5 assessments biweekly for at least 12 weeks. A total of 70% of app users completed the self-report of medication assessment daily for more than 100 days.” (lines 334-337)
	6aiii	9	**Describe whether, how, and when qualitative feedback was obtained from participants**<br>“After the 18-week end-of-study visit, patients and providers were invited to participate in a remote, qualitative, semistructured interview using a digital tool to discuss sentiments on app features and future features.” (lines 232-234)<br><br>“At the end of the long-term follow-up phase of the study (at year 1), a phone interview was conducted (34 weeks after use of the mobile app was discontinued) to assess patient-reported outcomes,..” (lines 238-241)
	6b	n/a	**“Any changes to trial outcomes after the trial commenced, with reasons”**<br>n/a
7	7ai	9	**“Describe whether and how expected attrition was taken into account when calculating the sample size”**<br>“This was a pilot study, and thus no sample size estimation was conducted. A sample size of 20 patients per group was expected to be sufficient to provide initial information about potential effects and benefits of the app and the feasibility of its real-world use to inform future larger-scale studies.” (lines 250-253)
	7b	n/a	**When applicable, explanation of any interim analyses and stopping guidelines**<br>n/a
8	8a	6	**“Method used to generate the random allocation sequence NPT: When applicable, how care providers were allocated to each trial group”**<br>“Participating patients were randomized based on the results from a randomized study list created using serially generated random numbers obtained by study staff using a random number generator (random.org).” (lines 144-146)
	8b	n/a	**“Type of randomisation; details of any restriction (such as blocking and block size)”**<br>n/a
9		6	**“Mechanism used to implement the random allocation sequence (such as sequentially numbered containers), describing any steps taken to conceal the sequence until interventions were assigned”**<br>“Participating patients were randomized based on the results from a randomized study list created using serially generated random numbers obtained by study staff using a random number generator (random.org).” (lines 144-146)

		Page #	Response
10		6	**“Who generated the random allocation sequence, who enrolled participants, and who assigned participants to interventions”**<br>”Upon identifying a patient with MDD who met criteria for study participation, the physician providing care for that patient (or a coordinator acting on behalf of the physician) explained the study, answered questions about the study, and obtained informed consent. Patients were then randomized to receive either the Pathway mobile app along with usual care or usual care alone. Participating patients were randomized based on the results from a randomized study list created using serially generated random numbers obtained by study staff using a random number generator (random.org).” (lines 140-146)
11	11ai	8	**“Specify who was blinded, and who wasn’t”**<br>**“**Eligible patients were randomized to usual care (n=20) or to usual care plus the mobile app (n=20) for 18 weeks (unblinded for both participants and researchers).” (lines 193-194)
	11aii	8	**Informed consent procedures (4a-ii) can create biases and certain expectations - discuss e.g., whether participants knew which intervention was the “intervention of interest” and which one was the “comparator.”**<br>**“**Eligible patients were randomized to usual care (n=20) or to usual care plus the mobile app (n=20) for 18 weeks (unblinded for both participants and researchers).” (lines 193-194)
	11b	n/a	**“If relevant, description of the similarity of interventions”**<br>n/a
12	12ai	10	**“Imputation techniques to deal with attrition / missing values”**<br>“Because 18-week follow-up data were not available for 3 randomized patients, these patients were dropped from the analysis, and an ITT analysis (with exclusion of missing data) was conducted on the remaining population. For the primary and long-term follow-up periods, between-group differences in changes in continuous variables were evaluated using a Student *t* test or Mann-Whitney *U* test.” (lines 254-258)
	12b	n/a	**“Methods for additional analyses, such as subgroup analyses and adjusted analyses”**<br>n/a
13	13a	10	**“Participant flow (a diagram is strongly recommended)”**<br>“Figure 3”<br><br>**“For each group, the numbers of participants who were randomly assigned, received intended treatment, and were analysed for the primary outcome”**<br>“A total of 40 patients were enrolled, and 92.5% (n=37) completed the 18-week primary follow-up period (Figure 3) and were included in the main analysis based on treatment allocation. In the app arm, 18 patients completed the primary follow-up period, 1 withdrew, and 1 was lost to follow-up. In the usual-care arm, 19 patients completed the primary follow-up period and 1 was lost to follow-up.” (lines 275-279)
	13b	10	**“For each group, losses and exclusions after randomization together with reasons”**<br>“In the app arm, 18 patients completed the primary follow-up period, 1 withdrew, and 1 was lost to follow-up. In the usual-care arm, 19 patients completed the primary follow-up period and 1 was lost to follow-up. At year 1 (the

		Page #	Response
			long-term follow-up phase), data were available for 17 patients (42.5%), including 8 patients in the mobile app arm and 9 in the usual-care arm" (lines 276-280)
14	14a	6	**"Dates defining the periods of recruitment and follow-up"**<br>"The study took place between July 2017 and January 2019 and involved 4 study sites in suburban and urban settings..." (lines 136-139)
	14b	n/a	Why the trial ended or was stopped [early]<br>n/a
15	15i	11-12	**"A table showing baseline demographic and clinical characteristics for each group"**<br>Table 1
16	16i	10	**"Report multiple "denominators" and provide definitions"**<br>"A total of 40 patients were enrolled, and 92.5% (n=37) completed the 18-week primary follow-up period (Figure 3) ... in the usual-care arm" (lines 275-280)
	16ii	10	**"Primary analysis should be intent-to-treat; secondary analyses could include comparing only "users", with the appropriate caveats that this is no longer a randomized sample"**<br>"Because 18-week follow-up data were not available for 3 randomized patients, these patients were dropped from the analysis, and an ITT analysis (with exclusion of missing data) was conducted on the remaining population." (lines 254-256)<br><br>"In the app arm, 18 patients completed the primary follow-up period, 1 withdrew, and 1 was lost to follow-up. In the usual-care arm, 19 patients completed the primary follow-up period and 1 was lost to follow-up. At year 1 (the long-term follow-up phase), data were available for 17 patients (42.5%), including 8 patients in the mobile app arm and 9 in the usual-care arm" (lines 276-280)
17	17ai	12-13	**"For each primary and secondary outcome, results for each group, and the estimated effect size and its precision (such as 95% confidence interval)"**<br>"At week 18, both arms exhibited an increase in patient activation based on PAM-13 score (Table 2), with greater improvement in the app arm than in the usual-care arm, although this difference was not statistically significant (mean change from baseline [SD]: 10.5 …: 1.7 [2.7] vs 0.6 [3.1]; *P*=.27)." (lines 303-309)<br><br>**"In addition to primary/secondary (clinical) outcomes, the presentation of process outcomes such as metrics of use and intensity of use (dose, exposure)"**<br>n/a
	17b	n/a	**"For binary outcomes, presentation of both absolute and relative effect sizes is recommended"**<br>n/a
18	18i	15	**"Results of any other analyses performed, including subgroup analyses and adjusted analyses, distinguishing pre-specified from exploratory"**<br>"A total of 2 patients in the app… these features during the next iteration" (lines 331-344)

		Page #	Response
			**A subgroup analysis of comparing only users is not uncommon in ehealth trials, but if done it must be stressed that this is a self-selected sample and no longer an unbiased sample from a randomized trial (see 16-iii).**<br>n/a
19	19i	15	**“All important harms or unintended effects in each group”**<br>**“Include privacy breaches, technical problems”.**<br>”One SAE (inpatient hospitalization related to depression) was reported in the app arm.” (line 332)
	19ii	15	**“Include qualitative feedback from participants or observations from staff/researchers, if available”**<br>“In remote interviews, more than 70% of patients and PCPs provided positive feedback on most of the app’s features, including its ability to track medication use and side effects, and provide reports” (lines 340-343)
20	20i	19	**Discussion**<br>**Trial limitations, addressing sources of potential bias, imprecision, and, if relevant, multiplicity of analyses**<br>**“Typical limitations in ehealth trials: participants in ehealth trials are rarely blinded. Ehealth trials often look at a multiplicity of outcomes, increasing risk for a Type I error. Discuss biases due to non-use of the intervention/usability issues, biases through informed consent procedures, unexpected events”**<br>“Potential limitations of… other platforms.” (lines 467-480)
21	21i	18-19	**“Generalizability to other populations”**<br>“Several systematic reviews... discontinuation rates observed in psychotherapy.” (lines 431-448)<br><br>“Moreover, approximately 60% of the study population was Hispanic, non-Hispanic Black, or multiracial, suggesting that the patients included in the study were largely representative of the racial and ethnic diversity observed in the US population.” (lines 461-464)
	21ii	19-20	**“Discuss if there were elements in the RCT that would be different in a routine application setting”**<br>“Additional work on the Pathway platform informed by the results of this study will help integrate the MDD digitally enabled care pathway into the current AAH system by assessing process and workflow improvements, clinician/ patient experiences, collaborative care model enhancements, EMR integration, and efficiencies with other platforms” (lines 476-480)
22	22i	16-18	**<u>Results</u>**<br>**“Interpretation consistent with results, balancing benefits and harms, and considering other relevant evidence Restate study questions and summarize the answers suggested by the data, starting with primary outcomes and process outcomes”**<br>“Results from this pilot study suggest that the Pathway mobile… the burden of in-person office visits” (lines 374-429)
	22ii	19-20	**“Highlight unanswered new questions, suggest future research”**<br>“The provision of additional education… other platforms” (lines 472-480)

		Page #	Response
			"The impact of the app on patient activation and MDD management will be further explored in a larger prospective study of its real-world use in patients with MDD" (ln 501-<br>502)
23		3	**"Registration number and name of trial registry"**<br>Clinical Trials.gov NCT03242213 (line 61)
24		21	**"Where the full trial protocol can be accessed, if available"**<br>"The data supporting the findings of this study are included within the published article (and its supplementary information files). The authors may be contacted for further data sharing." (lines 537-538)
25		21	**"Sources of funding and other support (such as supply of drugs)"**<br>"This study was funded by Takeda Pharmaceuticals U.S.A., Inc., and Lundbeck LLC. Medical writing assistance was provided by Nicole Cooper, on behalf of Syneos Health, and supported by Takeda Pharmaceuticals U.S.A., Inc., and Lundbeck LLC." (lines 517-519)
26	X26i	6	**"Comment on ethics committee approval"**<br>"This study was approved by the Advocate Health Care institutional review board." (lines 139-140)
	X26ii	6	**Outline informed consent procedures**<br>"Upon identifying a patient with MDD who met criteria for study participation, the physician providing care for that patient introduced the study; a designated research study coordinator then explained the study and obtained informed consent." (lines 140-143)
	X26iii	7	**Safety and security procedures**<br>"Patients reporting any change in suicidal ideation during PHQ-9 assessments were instructed to contact their health care provider or emergency services immediately since data from the app was not monitored; these instructions were reviewed with patients during the consent process." (160-163)
	X27	1, 5, 21	**"In addition to the usual declaration of interests (financial or otherwise), also state the "relation of the study team towards the system being evaluated" [2], i.e., state if the authors/evaluators are distinct from or identical with the developers/sponsors of the intervention"**<br>Author affiliations (lines 9-15)<br><br>"Takeda, Lundbeck, and Advocate Aurora Health (AAH) worked together with software developers (Ctrl Group/Fora Health & Cognition Kit) to develop the patient app and care team view of the patient data." (lines 112-114)<br><br>"MM contributed to the study design, data interpretation... critical review and approval of the manuscript." (lines 522-533)
